# Hypoglycemia Is Independently Associated with Multidimensional Impairment in Elderly Diabetic Patients

**DOI:** 10.1155/2014/906103

**Published:** 2014-02-13

**Authors:** A. Pilotto, M. Noale, S. Maggi, F. Addante, A. Tiengo, P. Cavallo Perin, G. Rengo, G. Crepaldi

**Affiliations:** ^1^Geriatrics Unit, Azienda ULSS 16, S. Antonio Hospital, 35127 Padua, Italy; ^2^CNR—Institute of Neuroscience, Aging Branch, Via Giustiniani 2, 35128 Padua, Italy; ^3^Geriatrics Unit, IRCCS Casa Sollievo della Sofferenza, San Giovanni Rotondo, 71013 Foggia, Italy; ^4^Department of Medicine, University of Padua, 35128 Padua, Italy; ^5^Diabetology Unit, University of Turin, 10124 Turin, Italy; ^6^Salvatore Maugeri Foundation, IRCCS, Scientific Institute of Telese Terme, 82037 Benevento, Italy

## Abstract

*Aim*. To identify the characteristics associated with multidimensional impairment, evaluated through the Multidimensional Prognostic Index (MPI), a validated predictive tool for mortality derived from a standardized Comprehensive Geriatric Assessment (CGA), in a cohort of elderly diabetic patients treated with oral hypoglycemic drugs. *Methods and Results*. The study population consisted of 1342 diabetic patients consecutively enrolled in 57 diabetes centers distributed throughout Italy, within the Metabolic Study. Inclusion criteria were diagnosis of type 2 diabetes mellitus (DM), 65 years old or over, and treatment with oral antidiabetic medications. Data concerning DM duration, medications for DM taken during the 3-month period before inclusion in the study, number of hypoglycemic events, and complications of DM were collected. Multidimensional impairment was assessed using the MPI evaluating functional, cognitive, and nutritional status; risk of pressure sores; comorbidity; number of drugs taken; and cohabitation status. The mean age of participants was 73.3 ± 5.5 years, and the mean MPI score was 0.22 ± 0.13. Multivariate analysis showed that advanced age, female gender, hypoglycemic events, and hospitalization for glycemic decompensation were independently associated with a worse MPI score. *Conclusion*. Stratification of elderly diabetic patients using the MPI might help to identify those patients at highest risk who need better-tailored treatment.

## 1. Introduction

The prevalence of diabetes mellitus (DM) is continuously rising worldwide especially among the older individuals. The prevalence rates range from 15% to 18.5%, about 12 times higher than the prevalence among younger people (under 45 years of age) [1, 2].

The elderly population exhibits widely heterogeneous clinical and functional health status, ranging from successful aging to frailty. Frailty has been identified as the most powerful predictor of mortality in the elderly population [3]. Recently, an operational definition of frailty has emerged in relation to its applicability to clinical practice [4]. According to this model, frailty has been defined as a condition of increased risk for negative health outcomes, including hospitalization and mortality, related to the presence of multidimensional impairments in different domains, that is, biological, clinical, functional, psychological, and social [5]. Accordingly to this concept, the most appropriate methodology to detect frailty is the Comprehensive Geriatric Assessment (CGA) [6, 7]. Recently a Multidimensional Prognostic Index (MPI) derived from a standardized CGA has been developed and validated in several independent cohorts of hospitalized [8] and community-dwelling [9] elderly patients. The good accuracy and calibration of the MPI as predictive tool for mortality have been recently confirmed by independent reviews and meta-analysis [10, 11]. Moreover, the MPI demonstrated a significant higher predictive power for short- and long-term all-cause mortality than other frailty instruments in a multicentre study on hospitalized older patients [12].

DM provides a clear example of a significant interaction between an organ disease and multidimensional impairments. Indeed, it has been reported that DM is associated with significant higher risk of disability [13, 14] and cognitive impairment [15]. Moreover, DM is often associated with depression [16], malnutrition [17], and increased risk of falls [18, 19] in the elderly population. On the other hand, it is well known that functional autonomy, cognitive function, social status, comorbidities, polypharmacotherapy, and finally life expectancy in the elderly population may influence treatment decisions. Therefore, identification of factors associated with multidimensional impairment would be very useful to program prevention strategies of frailty. Thus, the purpose of the present study was to identify the characteristics associated with multidimensional impairment, as evaluated by the CGA-based MPI, in a cohort of elderly diabetic patients treated with oral hypoglycemic drugs.

## 2. Methods

### 2.1. Study Population

All analyses were performed utilizing data from the Metabolic Study, a multicentre cross-sectional survey conducted in Italy, described in detail elsewhere [20]. Patients were consecutively enrolled in 57 diabetes centers distributed throughout Italy between September 2010 and October 2011. Participating centers were identified by the members of the Steering Committee, based on their previous involvement in research studies on diabetes. To be included in the study, patients needed to fulfill the following criteria: age ≥ 65 years, diagnosis of type 2 DM, treatment with oral antidiabetic drugs (with no changes in therapy in the three months before inclusion in the study), and the capacity to consent to the study protocol. The study was carried out in accordance with the guidelines for the classification of observational studies on drugs (Italian Medicines Agency—AIFA—determination on March 20, 2008—Gazzetta Ufficiale n. 76, March 31, 2008). All patients gave written informed consent and the Ethical Committee of each participating center approved the protocol.

### 2.2. The Metabolic Questionnaire

The Metabolic questionnaire was administered by the Diabetologists and collected cross-sectional data on

- patients' social history (living status; formal and informal healthcare);
- history of diabetes (duration; medications taken during the precedent three months);
- hypoglycemic events during the preceding three months (i.e., episodes characterized by autonomic symptoms such as tremor, hunger, sweating, and palpitations and nonspecific symptoms such as headache and nausea) reversed with the administration of sugar;
- drug therapy (excluding antidiabetic medications);
- assessment of the global health status using the multidimensional prognostic index (MPI [8]).

Blood pressure and heart rate were measured at the end of the interview.

Diabetic complications (coronary, cerebrovascular, peripheral arteriopathy, nephropathy, retinopathy, and neuropathy) and hematochemical determinations (referring to the precedent six months) were available in the clinical records and were recorded in the questionnaire form by the diabetologists. Blood samples were collected from each patient prior to completing the METABOLIC questionnaire (mean time: 11 days before completing the questionnaire).

### 2.3. The Multidimensional Prognostic Index

The multidimensional impairment of the patients enrolled in the study was evaluated by the MPI based on a standardized CGA that included information on basal and Instrumental Activities of Daily Living (ADL, IADL), the cognitive status assessed by the Short Portable Mental Status Questionnaire (SPMSQ), the risk of pressure ulcers evaluated by the Exton-Smith scale, and the nutritional status evaluated by the Mini Nutritional Assessment (MNA). Information on comorbidity, evaluated by the Cumulative Illness Rating Scale (CIRS), the number of medications, and the cohabitation status were also collected. From all these domains of the CGA, the MPI, a multidimensional predictive tool for short- and long-term mortality risk, was calculated according to a validated algorithm [8]. The final score of the MPI ranges from 0 (lowest risk) to 1 (highest risk). As previously reported [8], for clinical purposes three grades of MPI severity were identified according to well-defined cutoffs in order to stratify the examined population into three groups of multidimensional impairment risk: low risk (MPI-1, values ≤ 0.33), moderate risk (MPI-2, values between 0.34 and 0.66), and severe risk (MPI-3, values > 0.66) of mortality.

### 2.4. Statistical Analysis

The data are shown as means ± standard deviation or median for quantitative measures and frequency percentages for all discrete variables. MPI score was dichotomized into “low risk” (i.e., MPI value ≤ 0.33) versus “moderate or severe risk” (i.e., MPI value from 0.34 to 1.0). The differential distribution of the characteristics measured by the questionnaire in relation to MPI score dichotomized was assessed using the *χ* ^2^ test or Fisher's exact test for categorical variables (alpha = 0.05, two tail). Quantitative variables were compared utilizing the Generalized Linear Models (GLM) after verifying the homoschedasticity (Levene's test; in the event of heteroschedasticity Welch's ANOVA was considered) or the nonparametric Mann-Whitney test.

Logistic regression models were defined to identify characteristics associated with moderate/severe risk MPI grade. The independent variables considered in the models regard clinical characteristics (hematochemical parameters; hypoglycemic events during the preceding three months; diabetic complications such as coronary, cerebrovascular, peripheral vascular, nephropathy, retinopathy, neuropathy, and diabetes duration), anthropometric evaluations (Body Mass Index (BMI) and arm, calf, and waist circumferences), as well as sex and age. The identification of any characteristic associated with a moderate/severe risk of MPI was conducted considering, first, univariate logistic regression; significant variables with *P* ≤ 0.20 were introduced into a multivariate model to select variables associated using the backward selection method. Linearity assumption was evaluated for quantitative variables considering the analysis of quartiles. Odds ratio (OR) and corresponding 95% confidence intervals (95% CI) were calculated for each associated characteristic. A logistic model was constructed following the same statistical procedure to identify factors associated with hypoglycemic events.

The analyses were carried out using SAS software 9.2.

## 3. Results

### 3.1. Study Population

We enrolled 1342 consecutive patients with a diagnosis of type 2 DM. Mean age of patients was 73.3 ± 5.5 years and 52.5% were male patients. The mean duration of DM was 11.3 ± 8.2 years. At the time of enrollment, 50.1% of patients were treated with sulfonylureas, 29.7% with biguanides, 6.2% with dipeptidyl peptidase-4 (DPP-4) inhibitors, and 9.7% with insulin in association with oral hypoglycemic medications. One hundred sixty-one patients (12.0%) reported at least one hypoglycemic event in the three months before enrollment, requiring clinical assistance in 30 cases (18.6%). Almost all hypoglycemic events (142 patients, 88.2%) occurred in patients treated with insulin plus oral drugs (24.2%) or sulfonylureas (64%). Hypoglycemic events were also reported by 14 patients treated with biguanides, 3 patients treated with DPP-4 inhibitors plus oral drugs, and in one patient treated with alpha-glucosidase inhibitors (Table 1). Factors associated with hypoglycaemic events in a logistic regression model were insulin and sulphonylureas (Odds Ratio (OR) 8.82, 95% CI 4.47–17.40; *P* < 0.0001 and OR 4.79, 95% CI 2.70–8.49; *P* = 0.0168, resp.), diabetes duration in years (OR 1.04, 95% CI 1.02–1.06; *P* = 0.0002) and MNA score (OR 0.85, 95% CI 0.80–0.90; *P* ≤ 0.0001).

### 3.2. Characteristics of Patients Stratified According to the MPI Score

The mean MPI score was 0.22 ± 0.13 in the overall study population. After patient stratification according to MPI score, 1153 (85.9%) patients were in the MPI low risk group (MPI group 1), 180 (13.4%) patients in the MPI moderate-risk group (MPI group 2), and 9 (0.7%) in the MPI severe risk group (MPI group 3). For analyses, patients in the MPI 2 (moderate risk) and 3 (severe risk) groups were considered as a single group (moderate/severe risk). Demographic, clinical, and biochemical characteristics of patients divided according to the severity of multidimensional impairment are reported in Table 1. Patients in moderate/severe MPI group were older, mainly women, and had a longer average duration of diabetes (in years), higher prevalence of major macrovascular and microvascular complications, higher amount of hospitalization due to hypoglycemia or glycemic decompensation compared with patients included in the MPI low risk group. Moreover, a moderate/severe MPI score identified those patients treated with higher number of oral hypoglycemic drugs and with higher creatinine and triglycerides levels compared to patients in the MPI lower risk group. No statistically significant differences between the two groups were found for heart rate and blood pressure, fasting blood glucose, total cholesterol, and glycosylated hemoglobin levels.

### 3.3. Factors Associated with Moderate/Severe Multidimensional Impairment

Table 2 shows the demographic and clinical characteristics associated with a moderate/severe multidimensional impairment, as evaluated by the MPI. As expected, at multivariate analysis, older age and female gender were significantly associated with MPI higher risk groups. Interestingly, the prevalence of hypoglycemic events, hospitalization rates for glycemic decompensation, and a gained weight during the last three months were also significantly associated with moderate/severe risk groups of the MPI. Moreover, patients with overweight, defined as a BMI between 25 and 30 kg/m^2^, demonstrated an inverse significant association with the severity of MPI (OR = 0.59, 95% CI 0.36–0.97).

## 4. Discussion

This study demonstrates that in elderly type 2 diabetic patients older age, female gender, hypoglycemic events, and hospitalization due to diabetes decompensation are significantly correlated to the severity of multidimensional impairment, as assessed by the MPI. In this population, 12% of patients reported one or more episodes of hypoglycemia in the previous three months to the inclusion in the study. This observation is in agreement with data of Bramlage et al. [21] who reported a rate of 10.7% of hypoglycemic events within the 12 months prior to study inclusion in elderly diabetic patients with a direct correlation between age and number of hypoglycemic events. Recently, it has been reported that older patients with DM showed a significant higher risk of developing hypoglycemia probably related to the presence of several predisposing factors such as comorbidity, polypharmacy, chronic renal or hepatic impairment, poor nutrition, and altered counter-regulatory and symptomatic responses to hypoglycemia [22]. Data of this study confirmed that hypoglycemic events occurred significantly more frequently in patients included in the moderate/severe MPI risk groups; that is, more frail patients, who were affected from a significantly higher prevalence of major macrovascular and microvascular complications, were more underweighted or obese and with higher mean creatinine values compared to patients included in the mild MPI risk group. Interestingly, mean fasting blood glucose and glycosylated hemoglobin levels were not different between the different MPI groups, indirectly suggesting that hypoglycemic events should be more frequent in the frail elderly patients because of their altered symptom profile and/or altered glycemic thresholds. It has been reported that hypoglycemia in elderly patients is associated with an increased risk of falls and fractures resulting in the loss of functional independence and poor quality of life. Additionally, hypoglycemia is associated with an increased prevalence of cognitive impairment, higher hospitalization rates, and longer hospital stay. To the best of our knowledge, this is the first study that demonstrates that hypoglycemia is a characteristic associated with multidimensional impairment, that is, frailty, defined, in agreement to a current operational definition [4, 5], as a condition of increased risk for negative health outcomes, including mortality, related to the presence of multidimensional impairments in different domains. Conversely, it is also possible that frail individuals were more prone to hypoglycemia. The cross-sectional nature of the study, however, does not allow any conclusion regarding the causality of the association between hypoglycemia and worse MPI score.

The findings of this study reinforce the importance of preventing hypoglycemic events in the elderly population by means of a personalized treatment. In fact, in this study, the finding that hypoglycemic events in the 3-month period before study inclusion were significantly associated with higher MPI score, a good predictor of short- and long-term mortality in elderly patients with several clinical conditions [23], could indicate an higher mortality risk in diabetic patients who experienced hypoglycemic events.

In this contest, in agreement with the current view suggesting an important role of prognosis for the therapeutic decision-making process in the older patient with DM [24], the use of an accurate and well-calibrated tool to estimate multidimensional impairment of patients, such as MPI, might help to identify elderly patients at highest risk of mortality, independently of other markers of long-term glycemic control, that is, HbA1c levels. Indeed, in this study population, HbA1c was not correlated to multidimensional impairment.

Also an increase in hospitalization rate for diabetic decompensation has been observed in the elderly patients [25]. The geriatric population is at particular risk for developing hyperglycemic crises due to the age-related impairment of insulin secretory reserve, insulin sensitivity, and thirst mechanism; thus, elderly diabetic patients are particularly vulnerable to hyperglycemia and dehydration, the key components of hyperglycemic emergencies [26]. Additionally, in the elderly patients, disability and social factors can affect delivery of care and control [27]. Moreover, it is important to underlie that older patients with glycemic decompensation are less likely to have been using insulin before hospitalization and they tend to receive more insulin therapy during their hospital management, to have a longer duration of hospital stay and to have a higher mortality rate [28]. Thus, it is not surprisingly that hospitalization due to diabetic decompensation was independently associated with the severity of multidimensional impairment, supporting the predictive power of MPI score on negative outcome in the elderly.

Interestingly, in the present multicenter observational study overweight emerges to be inversely correlated to the multidimensional impairment. This finding is in agreement with previous data showing that in the elderly population overweight may be a protective factor for mortality in contrast to what occurs in young adult populations [29].

This study has two main limitations. First, a potential selection bias could have affected our sampling. Indeed, it is not possible to exclude that elderly patients with more severe functional disability, that is, with difficulty to access to ambulatory services for diabetes, may be more likely to refer to nonambulatory services. This may have led to an underestimation of multidimensional impairment in the elderly diabetic population. Secondly, hypoglycaemic events were recorded on an anamnestic basis where patients were required to recall events within the last 3 months.

In conclusion, we have demonstrated that age, female sex, hospitalization for glycemic decompensation, and hypoglycemic events are independently associated with multidimensional impairment, measured by MPI, in elderly diabetic patients. Since hypoglycemia is a potentially preventable predictor of multidimensional impairment, that in turn is associated with an higher mortality risk, these findings suggest to carefully take into consideration the episodes of hypoglycemia in the management and treatment of older diabetic patients.

## Figures and Tables

**Table 1:** Demographic and clinical characteristics of older patients with type 2 DM, overall and stratified according to the MPI risk.

	Overall (*n* = 1342)	MPI risk group		*P* value
		Low risk (*n* = 1153)	Moderate/severe risk (*n* = 189)	
Sex (male), *n* (%)	704 (52.5)	638 (55.3)	66 (34.9)	<0.0001
Age (years), mean ± SD	73.3 ± 5.5	72.9 ± 5.3	75.8 ± 6.1	<0.0001
Systolic blood pressure (mmHg), mean ± SD	138.3 ± 16.9	138.3 ± 16.8	138.6 ± 18.1	0.8404
Diastolic blood pressure (mmHg), mean ± SD	77.7 ± 8.6	77.8 ± 8.5	77.4 ± 9.2	0.6096
Heart rate, mean ± SD	74.3 ± 8.6	74.3 ± 8.5	74.3 ± 8.9	0.9727
Duration of diabetes (years), mean ± SD	11.3 ± 8.2	11.1 ± 8.1	12.4 ± 8.9	0.0399
Number of antidiabetic drugs, median	2 (1, 2)	1 (1, 2)	2 (1, 2)	<0.0001
Hypoglycemic events in the last 3 months, *n* (%)	161 (12.0)	122 (10.6)	39 (20.6)	<0.0001
Hospitalization for glycemic decompensation, *n* (%)	30 (2.2)	13 (1.1)	17 (9.0)	<0.0001
Gained weight, *n* (%)	256 (19.1)	202 (17.5)	54 (28.6)	0.0003
Diabetes complications, *n* (%)				
Coronary disease	256 (20.0)	194 (17.6)	62 (34.6)	<0.0001
Cerebrovascular disease	148 (11.3)	100 (8.9)	48 (26.8)	<0.0001
Peripheral arteriopathy	268 (20.7)	200 (17.9)	68 (37.8)	<0.0001
Nephropathy	212 (16.2)	159 (14.1)	53 (29.3)	<0.0001
Retinopathy	241 (18.6)	183 (16.3)	58 (33.0)	<0.0001
Neuropathy	188 (15.0)	138 (12.7)	50 (30.3)	<0.0001
Laboratory test				
Glycaemia, (mg/dL) mean ± SD	138.5 ± 35.4	138.7 ± 36.3	137.2 ± 29.9	0.5857
Creatinine, (mg/dL) median	0.90 (0.78, 1.10)	0.90 (0.70, 1.10)	0.98 (0.80, 1.30)	0.0014
HDL cholesterol, (mg/dL) mean ± SD	49.9 ± 12.9	49.9 ± 13.0	49.7 ± 12.7	0.8005
Triglycerides, (mg/dL) median	117.0 (88.0, 158.0)	115.0 (86.0, 154.0)	132.0 (98.0, 177.0)	<0.0001
HbA1c, (%) mean ± SD	7.1 ± 1.0	7.1 ± 1.1	7.2 ± 1.0	0.4413
BMI, *n* (%)				0.0008
Underweight (BMI < 18.5 kg/m^2^)	3 (0.2)	2 (0.2)	1 (0.5)	
Normal weight (BMI 18.5–25 kg/m^2^)	245 (18.3)	210 (18.2)	35 (18.5)	
Overweight (BMI 25–30 kg/m^2^)	550 (41.0)	495 (42.9)	55 (29.1)	
Obese (BMI ≥ 30 kg/m^2^)	544 (40.5)	446 (38.7)	98 (51.9)	
Antidiabetic therapies currently being taken, *n* (%)				
Insulin (in combination with other drugs including sulfonylureas)	62 (4.6)	42 (3.6)	20 (10.6)	<0.0001
Insulin (in combination with other drugs excluding sulfonylureas)	69 (5.1)	54 (4.7)	15 (7.9)	
Sulfonylureas (alone or in combination with other drugs excluding insulin)	672 (50.1)	570 (49.4)	102 (54.0)	
Only biguanides	399 (29.7)	368 (31.9)	31 (16.4)	
Only alpha-glucosidase inhibitors	12 (0.9)	9 (0.8)	3 (1.6)	
Only thiazolidinediones	12 (0.9)	10 (0.9)	2 (1.1)	
Biguanides and thiazolidinediones	31 (2.3)	27 (2.3)	4 (2.1)	
Dpp-4 inhibitors (alone or in combination with other drugs)	83 (6.2)	71 (6.2)	12 (6.4)	
Other associations	2 (0.2)	2 (0.2)	0 (0.0)	

**Table 2:** Demographic and clinical characteristics associated with a moderate/severe Multidimensional Prognostic Index (MPI) grade in 1342 older patients with type 2 DM.

	MPI Moderate/severe risk versus low risk		
	OR	95% CI	*P* value
Age (years)	1.11	1.07–1.14	<0.0001
Sex (female)	2.41	1.69–3.44	<0.0001
Hypoglycemic events in the last 3 months	1.83	1.17–2.86	0.0084
Gained weight in the last 3 months	1.84	1.24–2.74	0.0027
Hospitalization for glycemic decompensation in the last 3 months	7.67	3.32–17.7	<0.0001
Triglycerides (log transformed)	1.78	1.21–2.63	0.0037
BMI			0.0148
Underweight (BMI < 18.5 kg/m^2^)	0.98	0.06–15.9	
Normal weight (BMI 18.5–25 kg/m^2^)	1.00		
Overweight (BMI 25–30 kg/m^2^)	0.59	0.37–0.97	
Obese (BMI ≥ 30 kg/m^2^)	1.12	0.71–1.79	

## Acronyms

ADL: Activities of Daily Living
BMI: Body Mass Index
CGA: Comprehensive Geriatric Assessment
CI: Confidence Interval
CIRS: Cumulative Illness Rating Scale
DM: Diabetes mellitus
DPP-4: Dipeptidyl peptidase-4
GLM: Generalized Linear Models
IADL: Instrumental Activities of Daily Living
MNA: Mini Nutritional Assessment
MPI: Multidimensional Prognostic Index
OR: Odds Ratio
SPMSQ: Short Portable Mental Status Questionnaire.
